# Instructions Relative to Railroad Fares

**Published:** 1896-08

**Authors:** 


### Instructions Relative to Railroad Fares.

1. The reduction is to persons going to the meeting from territory of the Trunk-Line Association, and we hope we can say from the Missouri River the Atlantic Ocean.

2. The reduction is fare and one-third on committee's certificate, conditioned on there being an attendance at the meeting of not less than one hundred persons who have travelled thereto on some legitimate form of railroad transportation.

3. The reduction applies to persons starting from trunk-line territory by any of the roads named below, who have paid 75 cents or upward for their going journey. Each person availing of it will pay full first-class fare going to the meeting and get a certificate filled in on one side by the agent of whom the ticket is purchased. Agents at all important stations and coupon-ticket offices are supplied with certificates.

4. *Certificates are not kept at all stations.* If, however, the ticket agent at a local station is not supplied with certificates and through tickets to the place of meeting, he can inform the delegate of the nearest important station where they can be obtained. In such a case the delegate should purchase a local ticket to such station and there take up his certificate and through ticket to place of meeting.

5. Going tickets, in connection with which certificates are issued for return, may be sold only within three days (Sunday excepted) prior to and during the continuance of the meeting; except that when meetings are held at distant points to which the authorized limit is greater than three days, tickets may be sold before the meeting in accordance with the limits shown in regular tariffs.

6. Deposit the certificate with the Secretary or other proper officer of the organization at the meeting, for necessary indorsement and visé of special agent.

7. Certificates are not transferable, and return tickets secured upon certificates are not transferable.

8. On presentation of the certificate, duly filled in on both sides, within three days (Sunday excepted) after the adjournment of the meeting, the ticket agent at the place of meeting will return the holder to starting-point, by the route over which the going journey was made, at one-third the highest limited fare by such route. The return tickets will in all cases be closely limited to continuous passage to destination.

9. No refund fare will be made on account of any person failing to obtain a certificate.

